# Surgical-only treatment of pancreatic and extra-pancreatic metastases from renal cell carcinoma - quality of life and survival analysis

**DOI:** 10.1186/s12893-020-00757-0

**Published:** 2020-05-13

**Authors:** Stefania Brozzetti, Simone Bini, Nelide De Lio, Carlo Lombardo, Ugo Boggi

**Affiliations:** 1 Surgical Department “Pietro Valdoni”, Policlinico Umberto I, University of Rome “La Sapienza”, Viale del Policlinico 155, 00161 Rome, Italy; 2 Division of General and Transplantation Surgery, University of Pisa, Pisa, Italy

**Keywords:** Renal cell carcinoma, RCC, Pancreatic resection, Robotic surgery, Pancreatic metastases, Quality of Life; cost-effectiveness.

## Abstract

**Background:**

Treatment of pancreatic metastases (PM) from renal cell carcinoma (RCC) is still an issue between surgeons and oncologists, in the era of target-therapy.

**Methods:**

Data from 26 patients undergoing resection of PM and extra-PM from RCC, with R0 intention were retrospectively analysed. No one received adjuvant chemotherapy. Patients were divided into two groups; Group A comprehends 14 patients who developed synchronous (5) or methacronous (9) extra-PM. Group B comprehends 12 patients that developed PM only.

**Results:**

No intraoperative mortality was recorded. Complications occurred in 14 patients (53.8%), all but 2 (7.26%) were graded I and II according to Clavien-Dindo classification.

Recurrences occurred in 8 patients (30.8%), of whom, 5 (62.5%) were submitted for further resections in other sites.

Three-, five- and ten-year observed overall survival were respectively 88,5% [95%CI: 0,56 – 1,33], 76,9% [95%CI: 0,47 – 1,19] and 50% [95%CI: 0,20 – 1,03]. Disease-free survival was 65,4% [95%CI: 0,38 – 1,05], at 3 years, 57,7% [95%CI 0,323 – 0,952] at 5 years and 42,9% [95%CI 0,157 – 0,933], at 10 years. QoL analysis, through WHOQOL-BREF questionnaire, assessed at last available follow up revealed a mean score of 75,9 ± 11,6 on 100 points.

**Conclusion:**

Despite no significant differences in survival between patients affected by Pancreatic or Extra-Pancreatic metastases, PM patients seems to show better outcome when managed surgically. mRCC patients, eligible for radical metastasectomy, tend to have long survival rates, reduced recurrence rates and good QoL.

**Study registration:**

This paper was registered retrospectively in ClinicalTrials.gov with Identification number: NCT03670992.

## Background

Metastatic lesions in pancreas are extremely rare, accounting for less than 5% of pancreatic malignancies diagnosed in living patients. Pancreatic metastases are found more frequently at autopsy, being identified in up to 15% of patients with malignant disease. While resection of metastatic lesions in liver and lung has been well described and is generally accepted to improve survival, the optimal management of pancreatic metastases is not yet well accepted and there are still issues among clinicians [1]. Synchronous metastases from RCC occur in 25–30% and metachronous metastases in about 40% of patients. Biology of metastatic RCC is heterogeneous. Recurrences may present within 1 year from nephrectomy with rapid progression of disease, or in some other cases, tumour-free intervals of more than 20 years may be observed with a slow growth pattern, especially for pancreatic metastases [2]. Advancements in technology and surgical techniques have reduced operative risk in pancreatic surgery and therefore, allowed a more aggressive approach in patients with solitary PM from RCC. Pancreatic surgery in this clinical setting has already proved satisfactory results [1–4]. Controversies still exist regarding indications to surgical resection of metachronous and/or synchronous pancreatic multiple metastases and extra-PM from RCC [5–7]. Attention has been paid mainly to overall survival and disease-free survival, QoL after different forms of treatment has not been well analysed. We report cases of twenty-six consecutive patients who underwent extended pancreatic resections for metastases from clear cell RCC in order to achieve R0 resection. Patients with history of intra- and extra-abdominal metastases, either in first presentation or during follow-up, were enrolled in our study. In case complete surgical resection of pancreatic and/or extra pancreatic metastases was unachievable chemotherapy was indicated. To our knowledge, this is the first paper that gives special attention to patients’ QoL after surgery for clear cell carcinoma, in a such long follow-up period.

## Methods

Retrospective data was analysed from 26 patients submitted to pancreatic resection between August 2002 and November 2015. Inclusion criteria were: single or multiple pancreatic or extra pancreatic metastases from clear cell carcinoma (Table 1) and treatment non-associated with adjuvant chemotherapy. Patients that already received a previous pancreatic resection were also included. Data in this manuscript has been reported in line with the PROCESS criteria [8].

**Table 1:**
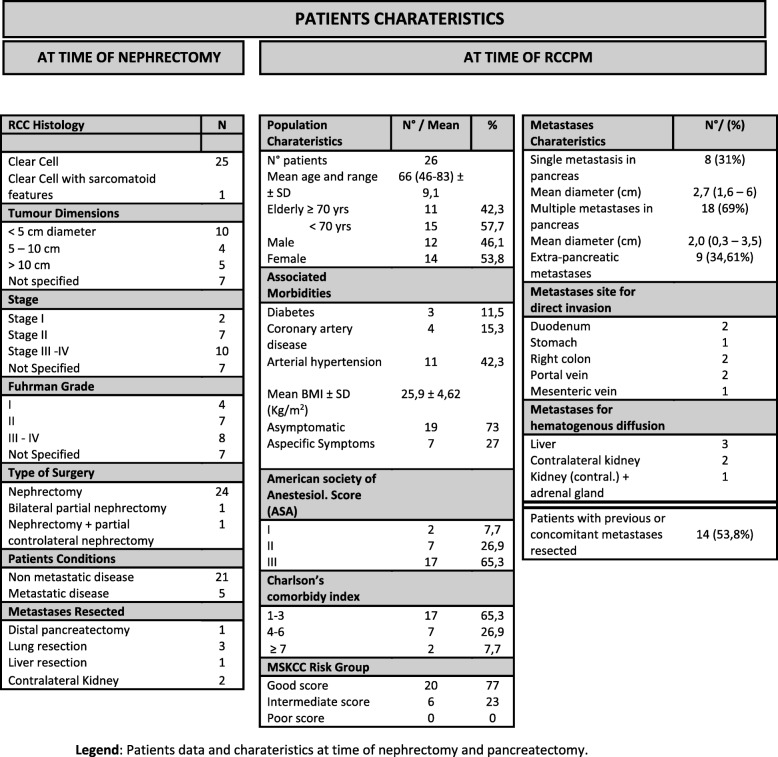
Patient's characteristics

Legend: Patients data and charateristics at time of nephrectomy and pancreatectomy.

Different kind of surgical approaches were taken into account in this study: duodenal-pancreatectomy, total-pancreatectomy and distal-pancreatectomy associated or not with other metastatic site resections. Surgery was performed either with classical open approach or modern robotic approach, using the robot Da Vinci® *Si* model. Aim of surgical interventions were to remove all metastases in association to radical lymphadenectomy thus to achieve R0 resection. Patients enrolled to cytoreductive surgery were excluded from this study and treated with adjuvant chemotherapy [5]. All postoperative events occurring within 90 days of surgery were considered. Postoperative complications were graded according to Clavien-Dindo classification. Patients were followed-up 3 months after discharge and every 6 months thereafter. Patients had blood chemistries and CT scans at least every year. MRI and bone scan were used in case of inconclusive CT-scan, in order to evaluate eventual liver, head and bone recurrences [5].

A database was used to record all patients’ data. Results were analysed in terms of Operative Mortality and Morbidity, Overall Survival, Disease-Free Survival and Quality of Life. Protocols approbation by the bioethical review committee was waived since the retrospective nature of the study and meet the guidelines of our institutions.

Patients were divided into two groups; Group A comprehends 14 patients who developed synchronous (5 recipients) or methacronous (9 recipients) extra-PM. Group B comprehends 12 patients that developed PM only.

Present QoL was measured through WHOQOL-BREF questionnaire [9] administered at last follow-up to the 19 patients still alive.

Retrospective QoL in past years was estimated through combination of different parameters and by questionnaires retrospectively fulfilled and based on patients’ recollection at one, three, five and ten years after surgery: Karnofsky performance scale, Activity of Daily Living scale (ADL) [10], Instrumental Activity of Daily Living scale (IADL) [11], and Geriatric Depression Scale (GDS) [12]. 

Nutritional status was measured by monitoring in clinical records weight loss through BMI, by serum albumin and haemoglobin levels at one, three, five and ten years after surgery: Hb > 12,5 g/dl one point, serum albumin > 2 g/dl one point, and BMI > 19,5 Kg/m^2 one point. Score 0–3.

Points were assigned through specific questionnaires to Karnofsky (Score 0–100), IADL (Score 0–8), ADL (Score 0–6) [10, 11].

As for ADL a score of 6 is considered as conserved daily activity; 4–5 as moderate impairment; 2–3 as mild impairment and 0–1 as severe impairment [10].

IADL measures impairment in instrumental activity, a score of 8 is considered a conserved instrumental activity, 4–7 as moderate impairment, 2–4 as mild impairment and 0–1 as severe impairment [11].

Presence or absence of depression was also considered and evaluated through Geriatric Depression Scale (GDS-15): depression cut-off was set at 5 points of the scale considering a score of 0–4 as non-depression; 5–9 as mild depression and 10–15 as severe depression [12].

QoL was defined by combination of these parameters as: excellent, good, fair, poor or very poor (Table 4).

Data was analysed via Chi-square test, as well as Mann-Whitney test for parametrical and non-parametrical values. Overall survival and disease-free survival were described by Kaplan-Meier analysis. A log-rank test was used to compare continuous variables and was expressed by survival curves. Statistical significance was set at *p* ≤ 0,05.

## Results

Twenty-six patients were submitted to nephrectomy for RCC, PM appeared in follow-up after median 13 years after primary tumour removal [range 2–35 years], except in one case where simultaneous resection of bilateral renal cancer and distal-pancreatectomy for metastases was performed. Eight patients had a single pancreatic metastasis, six of which greater than 2 cm in diameter.Eighteen patients had multiple pancreatic metastases, 10 of which greater than 2 cm in diameter.

Five patients underwent previous resection of lung metastases associated with liver metastases after nephrectomy (Table 1). Five patients, at time of pancreatic resection, had infiltration of one or more neighbouring organs. Six patients had haematogenous metastases (Table 1).

Overall, fourteen patients (53,8%) had previous or synchronous resected extra-PM. Patients were considered eligible for pancreatic resection by assessing ASA-score [13], Charlson’s age-adjusted comorbidity index [14] and the Memorial Sloan-Kettering Cancer Center risk score [15] that were used to stratify the population based on comorbidities and risk factors (Table 1).

Open surgery was performed in 22 patients; robotic surgery was performed in 4. Conversion rate robotic to open surgery was 0%. Complete resection was achieved in all cases, either in pancreatic and extra-pancreatic metastases, Table 2 describes the type of operations performed. Complete data on resected lymph nodes were available for 22 of the 26 patients. An average of 28 lymph nodes were resected. There was no difference between the average number of lymph nodes removed in the 4 patients who had robotic surgery (mean of 26 lymph nodes per patient) and the 18 who had open surgery (mean of 28 lymph nodes per patient). Out of the 659 lymph nodes analysed, only 5 nodes (0.7%) in 3 patients had cancer involvement.

Total pancreatectomy (TP) was performed in 13 patients; one underwent total pancreatectomy for recurrent pancreatic (uncus) metastases treated by left pancreatic resection 16 years earlier.

**Table 2:** Type of Surgery

TYPE OF SURGERY	
	N.
Single PM	8^a^
PD	5
DP + Liver resection + Portal vein resection and Thrombectomy	1
DP + Wedge resection contralateral kidney	1
TP + Resection and reconstruction mesenteric vein	1
Multiple PM	18^a^
TP	7
TP + Liver resection	1
TP + Injection alcohol liver meta- stases	1
TP + Contralateral nephrectomy + Adrenalectomy	1
TP + Right colon resection	1
TP + Portal vein resection + Right colon resection	1
PD	2
PD + Jejunal resection	1
DP	2
DP + Contralateral nephrectomy	1
DP + Nephrectomy + Contralateral wedge nephrectomy	1

*PD* Pancreatic Duodenectomy *DP* Distal Pancreatectomy

*TP* Total Pancreatectomy

^a^ Number of patients

Legend: Practiced surgical resections

Distal pancreatectomy (DP) was performed in 5 patients and pancreaticoduodenectomy (PD) in the remaining 8 patients. Spleen-preserving DP was performed in 2 of the 5 patients. The pylorus was spared in 17 of 21 patients who underwent TP or PD (81%). About the four robotic surgery procedures, 2 were PD, one was TP and one was DP. One patient underwent synchronous jejunum resection for a jejunal mass that revealed to be a GIST (Table 2). There was no operative mortality (within 90 days). Complications occurred in 14 patients (53.8%), all but two were grade I or grade II according to Clavien-Dindo classification and resolved rapidly.

One patient required re-laparotomy for intestinal bleeding, the other one had acute respiratory distress syndrome. Twelve patients with regular postoperative period left the hospital at an average of 14 days from the operation. Patients that underwent robotic surgery left the hospital 15,16, 23 and 30 days after surgery.

Hospitalization was more than 21 days in 7 patients due to pancreatic fistula (3 patients had biochemical leak; 2 Grade B, 0 Grade C) [16] and/or for the occurrence of delayed gastric emptying (2 patients Grade A; 2 Grade B; 2 Grade C) [17].

No patients were lost during follow-up. Follow-up ranged from 60 to 161 months (mean 104 months).

During follow-up, metastases, in distant sites from resected areas, recurred in 12 patients (46,2%) at an average of 34 months from pancreatic resection (range 15–70 months); 5 patients were suitable for a further resection. (Table 3).

**Table 3:**
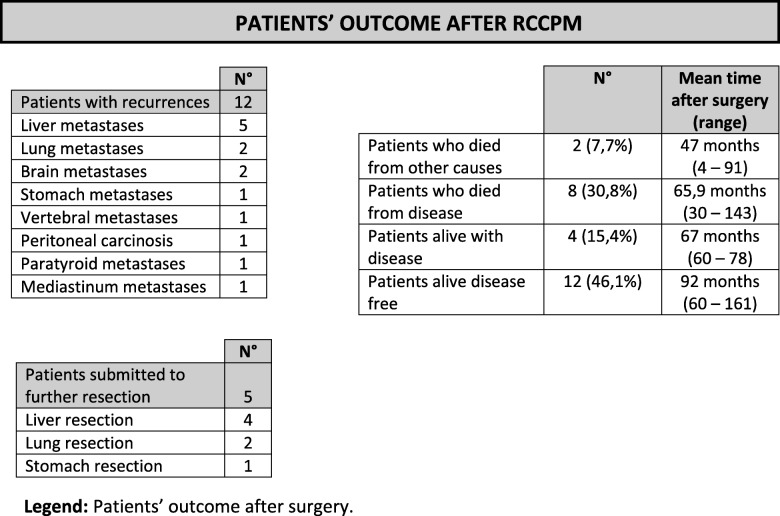
Patient's outcome

Legend: Patients’ outcome after surgery

Three cases (25%) came from the group with isolated PM and 5 cases (36%) from the group with extra-PM.

**Fig. 1:**
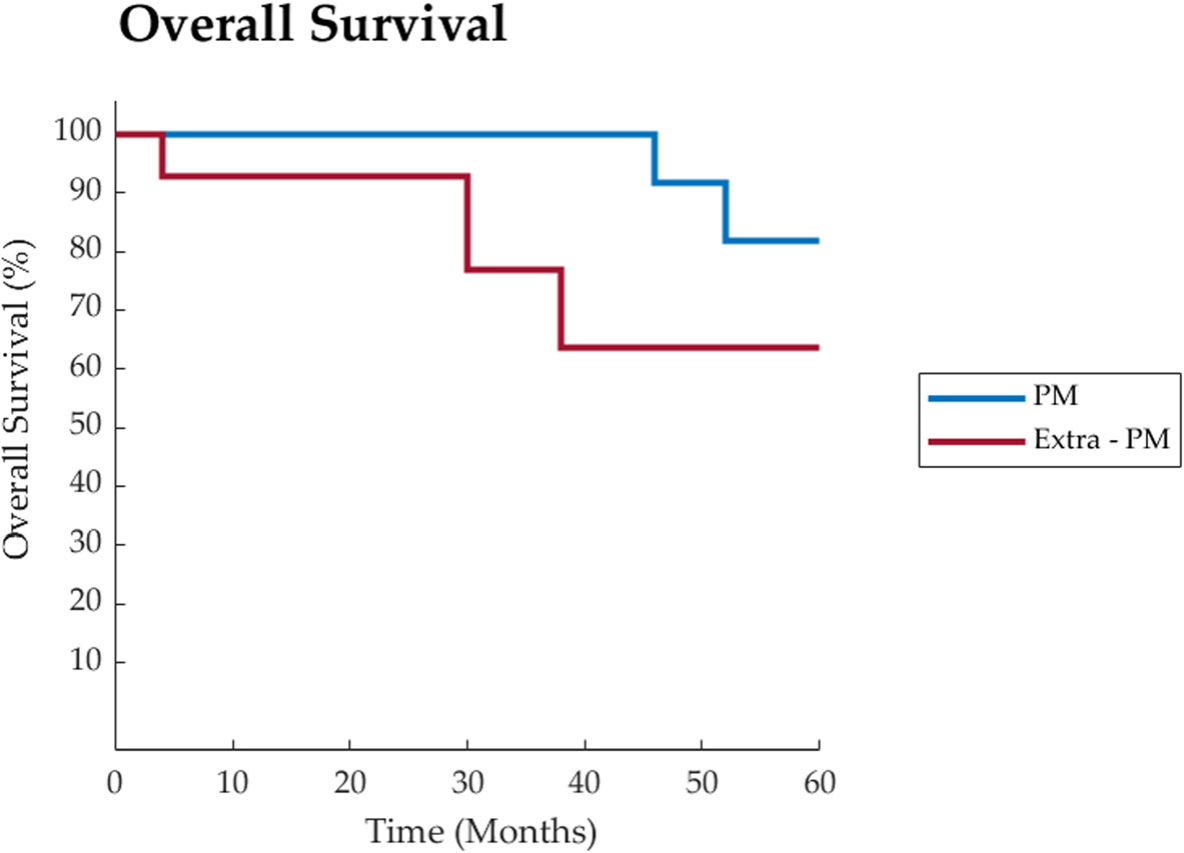
Overall Survival representation of PM group vs extra-PM group

Three-, five- and ten-year observed overall survival were respectively 88,5% [95%CI: 0,56 – 1,33], 76,9% [95%CI: 0,47 – 1,19] and 50% [95%CI: 0,20 – 1,03]. (Fig. 1).

**Fig. 2:**
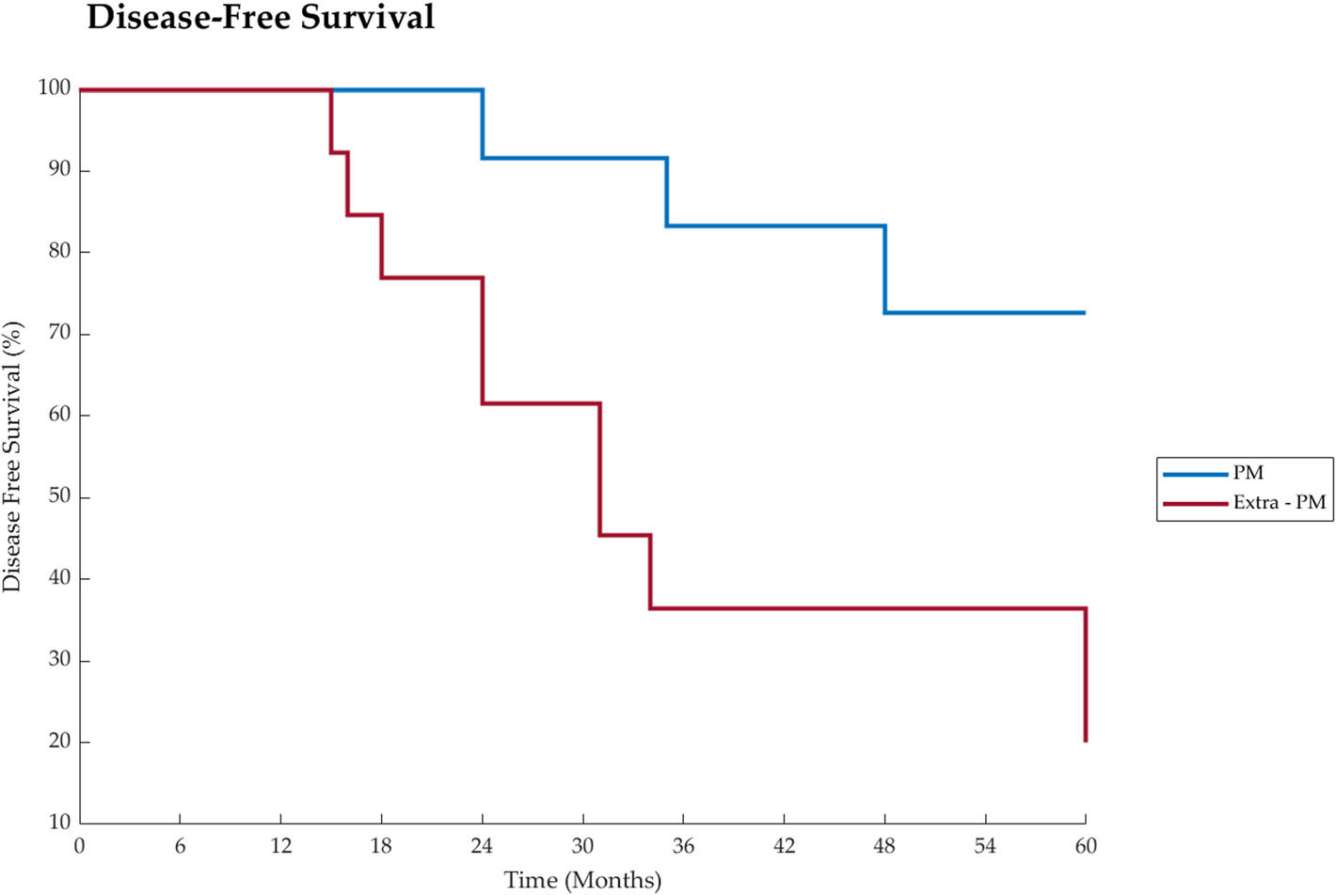
Disease free survival after surgery of PM group vs extra-PM group

Disease-free survival was 65,4% [95%CI: 0,38 – 1,05], at 3 years, 57,7% [95%CI 0,323 – 0,952] at 5 years and 42,9% [95%CI 0,157 – 0,933], at 10 years. (Fig. 2).

No specific risk factor influenced survival.

In Group A mean OS was 89,1 months ±52,4 SD [Range 4–162], mean DFS was 59,1 months ±52,9 SD [Range 4–162].

In Group B mean OS was 77,8 months ±24,5 SD [Range 23–141], mean DFS was 70,9 months ±31,1 SD [Range 23–141]. Mann-Whitney test revealed no statistically significant difference between the two groups either in OS and DFS (*p*_*OS*_ = 0,490 and *p*_*DFS*_ = 0,06).

Prevalence of recurrent metastatic disease (57,1% vs 33,3% [RR: 1,71; 95%CI: 0,459 – 7,7]) and tumour-related deaths (35,7% vs 16,7% [RR: 2,14; 95%CI: 0,351 – 22,5]) were higher in Group A, however no statistically significant difference was found in both cases (*p*_*RMD*_ = 0,149 and *p*_*TRD*_ = 0,099 respectively). Table 3 describes the status of the 26 patients at the end of the study period.

As for QoL analysis WHOQOL-BREF questionnaire was administered to 19 patients still alive, overall mean score was 75,9 on 100 points [± 11,6], dominion-related analysis is showed in Table 4.

**Table 4:**
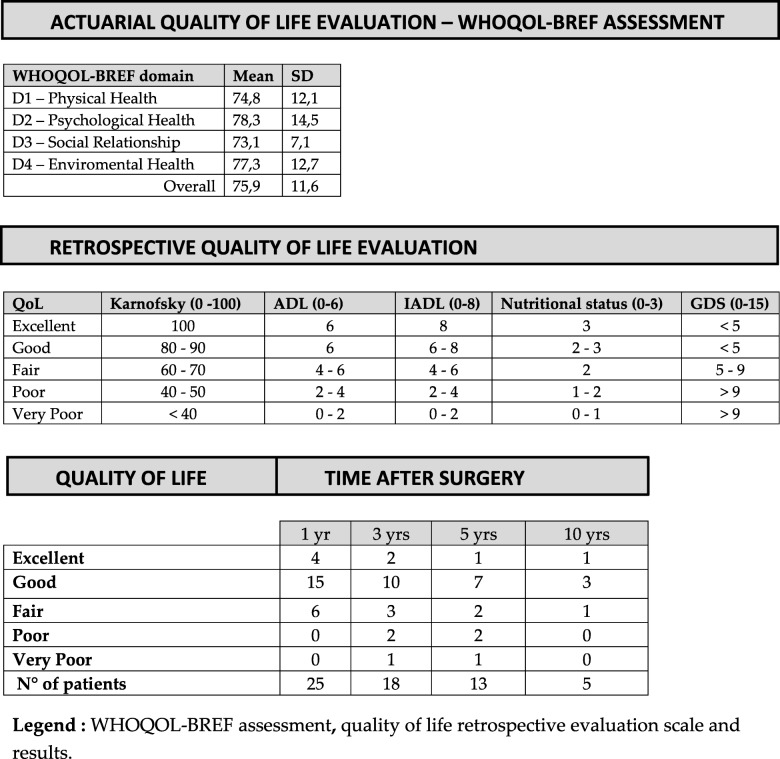
Quality of Life

Legend: WHOQOL-BREF assessment**,** quality of life retrospective evaluation scale and results

As for retrospective analysis 25 patients were alive one year after surgery, 18 were alive at three years, 13 at five years and 5 at ten years after surgery. Results of QoL analysis are displayed in Table 4.

All patients submitted to TP had pancreatic exocrine enzyme replacement therapy. Only two patients suffered from steatorrhea and remained respectively five and ten kilos below their preoperative weight, but blood chemistry did not show any evidence of malnutrition.

Three patients who had TP experienced episodes of hypoglycaemia in the first year after surgery, this was easily corrected without hospitalization.

## Discussion

Despite early diagnosis and treatment, one-third of patients suffering from clear cell RCC developed local or distant metastases [18].

Isolated PM from RCC are rare. Several reviews have reported satisfactory results after RCC’s PM excision [1–4]. *Adler* et al [1] reviewed 18 case-series: pancreatic metastases were observed mostly from RCC (62%); one and five-years survival rates were 86 and 50%respectively. All of them showed that excision of metastases from RCC had better OS, however extra PM were associated with poorer survival, not in line with our results. Hypothetically this difference is due to further resections that received our patients.

Surgical approach in metastatic disease is now an integral part of a multidisciplinary oncological therapy, good results have been reported after an aggressive removal of synchronous or methacronous metastases [19]. A complete resection of the tumour can be followed by unexpected long-term survival [1–4].

RCC is often associated with Von Hippel-Lindau (VHL) pathway mutation, which neutralizes the action of several hypoxia inducible factors (HIFs). Once active, HIFs lead to Vascular Endothelial Growth Factor (VEGF) activation that stimulates cell growth, vessel formation and leads to tumour aggressiveness. Recently, in several RCC was also shown an mTOR mutation that would boost cell growth and, therefore malignant transformation [20].

Target therapy for mRCC has been consistently developed in recent years, specific inhibitors of VEGF pathway, mTOR pathway and PD-1/PD-L1 showed the most consistent results. More than 11 molecules are available for mRCC treatment in first, second and consequent line protocols [21, 22]. (Table 5). Unexpected outcomes in terms of Objective Response were shown by latest investigated molecule combinations such as *Nivolumab/Ipilimumab* and *Atezolizumab/Bevacizumab* (Objective Response > 30%, but Complete Response < 10% and mean PFS of 13,1 and 10,4 months respectively) [22].

Many patients develop adverse effects that may be extremely debilitating (G3/G4 side effects). Intolerance to target therapy leads therefore to dose-reduction or therapy suspension, exposing recipients to disease progression’s risk [22]. Dose Reduction is needed in 40–50% of patients, Therapy Discontinuation in 20–25% and Treatment-Associated deaths are rare [22].

**Table 5:** Target therapy for mRCC

TARGET THERAPY FOR mRCC – SCHEMES & RESULTS				
Scheme/Drug	Line of Treatment	Molecular Target	Mean PFS (Months)	Mean OS(Months)
**Sunitinib**^1,2^	First	mTKI	11	26,4
**Temsirolimus**^1^	First	mTOR	1,9	10,9
**Pazopanib**^1^	First	mTKI	11,8	22,9
**Sorafenib**^1^	Second	mTKI	5,5	17,8
**Bevacizumab + IFNα**^1^	Second	VEGF-A	8,5	18,3
**Axitinib**^1^	Second	mTKI	6,7	20,1
**Everolimus**^1^	Second	mTOR	4	14,8
**Nivolumab**^1^	Second	PD-1	4,6	25
**Cabozatinib**^1,2^	Second	mTKI	7,4	21,4
**Lenvatinb + Everolimus**^1^	Second	mTKI/mTOR	14,6	NR
**Nivolumab + Ipilimumab**^2^	Second	PD-1/PD-L1	12,6	NR
**Atezolizumab + Bevacizumab**^2^	Second	PD-L1/VEGF-A	11,2	NR

Legend: *mTKI* Multiple Tyrosine Kinase Inhibitor**,***VEGF-A* Vascular Endotelial Growth Factor A, *mTOR* Mammalian Target of Rapamycin, *PD-1* Programmed Death 1, *PD-L1* Programmed Death Ligand 1, *NR* Not Reached

Most common adverse effects are: Hypertension, Fatigue, Mucositis, Diarrhoea, Neutropenia and Lymphopenia. *Nivolumab* and *Ipilimumab* are associated with immune mediated adverse effects in 80% of recipients [22]. For all these reasons, patients treated with target-therapy shall be followed-up very carefully, and therapeuthical adjustments shall be done according to individual patient’s response, that may be variable and unpredictable.

In mRCC, as this manuscript confirms, surgery remains an extremely valid option, although it suffers from an obvious selection bias, patients eligible to radical resection have longer DFS and reduced recurrence rate [1, 23, 24]. Robotic and Laparoscopic metastasectomy adds the possibility of a less traumatic surgical approach and widens surgical indications to patients previously considered unfit for open surgery [1, 7]. Moreover, radical surgery aims at complete removal of the malignancy while target therapy aims at controlling disease in a chronicised state. However, surgical resection of PMs may not always be the best treatment for RCC patients. Chemotherapy shall be preferred to pancreatic resection in stage IV RCC if complete resection is unachievable.

Surgery can also be cost-effective vs the most recent tyrosine-kinase inhibitor molecules that in a chronic therapy requires $ 20.000 and more per patient-year [25] vs a median of $ 40.000 per patient for surgery [26].

In our groups of patients we were not able to find significant differences in survival or disease recurrence among the two groups, however it is possible to deduce that in wider casuistic this may be possible, particularly in DFS and tumour recurrence in patients with only pancreatic metastases. Near-significant *p* values were found in comparing DFS (*p* = 0,06) and Tumour Related Deaths (*p* = 0,099) with better outcome for patients in Group B, therefore it is possible to consider patients with only Pancreatic Metastases from RCC, a long-surviving, less-progressing Group.

As for QoL analysis, WHOQOL-BREF questionnaire was administered to all patients alive at their last follow up, showed a mean score higher than 75 on 100, which is surprisingly high for metastatic patients.

QoL in past years might only be estimated retrospectively, in order to achieve this, we retained that WHOQOL-BREF was subject of too many biases, therefore we tried to focus on more objective parameters that could be easily recollected by patients although much time passed. In this retrospective research we aimed at focusing either on general physical performance evaluated by Karnofsky scale; patient’s ability to fulfil personal needs and to use daily instruments assessed through ADL and IADL scale; nutritional and psychological status. All these parameters are combined in a qualitative evaluation from very poor to excellent since quality of life is a multidimensional concept that includes one’s evaluation of well-being and functioning across physical, psychological, social, and sexual domains, with some conceptualizations including spiritual well-being [27].

In order to better evaluate patient’s QoL in surgery we decided to take some parameters from Comprehensive Geriatric Assessment (CGA) used in clinical oncology to assess patient’s QoL and fitness to chemotherapy [28], these parameters were chosen due to the experience matured in treating geriatric patients in context of our geriatric unit and with collaboration of the *Italian Geriatric Oncologic Group* (GOGI).

Quality of life is a critical outcome in clinical trials and a significant predictor of treatment response. In the existent PM’s surgical treatment studies, it is rarely or limitedly taken into account. Instead, this is one of few papers where QoL is carefully investigated. In spite of the small number of our recipients, many of them reported a good QoL, moreover, some report an active and sporty life for several years after surgery. Therefore, considering treatment for PM from RCC, surgery shall be preferred, especially in selected patients, anytime complete resection of RCC metastases is achievable either pancreatic and extra-pancreatic sites. Clear cell RCC is one the rare malignancies where surgery may achieve astonishing long DFS and OS with important QoL benefit. (Figs. 1 and 2).

## Conclusion

Clinical judgment remains of fundamental value in determining which therapeutic option is indicated for each patient. Considering results of surgical approach in single centre studies many questions remain unsolved, including indications for a possible complementary role of surgery and target therapy and in which cases metastatic dissemination is out of any possible surgical control. To our knowledge, complete resection in RCC metastases either pancreatic or extra pancreatic in fit patients, produce survival and QoL benefit when indolent growth patterns are showed by primary malignancy.

A multidisciplinary approach is essential, considering the patient as a whole in his/her individuality, with his/her own needs and expectations, therefore periodical QoL assessment and a lifelong follow-up are crucial in order to achieve patient’s wellbeing either in surgical or clinical settings. Patients with only Pancreatic Metastases seems to show a better outcome than Extra-Pancreatic Metastases’ patients, further and wider studies are necessary to better determine differences in survival and natural history of the disease observed by this manuscript, but prospective studies concerning this topic are difficult to run, considering the rarity and long natural evolution of the outlined disease.

## Data Availability

The datasets used and/or analysed during the current study are available from the corresponding author on reasonable request.

## Abbreviations

PM: Pancreatic Metastases
RCC: Renal Cell Carcinoma
mRCC: Metastatic Renal Cell Carcinoma
SD: Standard Deviation
QoL: Quality of Life
TP: Total Pancreatectomy
PD: Pancreaticoduodenectomy
DP: Distal Pancreatectomy
ADL: Activity of Daily Living
IADL: Instrumental Activity of Daily Living
GDS: Geriatric Depression Scale
BMI: Body Mass Index
CT: Computed Tomography
GIST: Gastric Interstitial Stromal Tumour
VHL: Von Hippel Lindau
HIF: Hypoxia Inducible Factor
VEGF: Vascular Endothelial Growth Factor
OS: Overall Survival
CI: Confidence Interval
PFS: Progression-Free Survival
DFS: Disease Free Survival
RR: Relative Risk
GOGI: Italian Oncologic Geriatric Group
CGA: Comprehensive Geriatric Assessment
RMD: Recurrent Metastatic Disease
TRD: Tumour-Related Deaths
